# The development and evaluation of the Australian child and adolescent recommended food score: a cross-sectional study

**DOI:** 10.1186/1475-2891-11-96

**Published:** 2012-11-19

**Authors:** Skye Marshall, Jane Watson, Tracy Burrows, Maya Guest, Clare E Collins

**Affiliations:** 1 Lighthouse Nutrition Pty Ltd, 1/23 Namitjira Place, Ballina, 2478, NSW, Australia; 2 Nutrition and Dietetics, School of Health Sciences, Faculty of Health, The University of Newcastle, Callaghan, 2308, NSW, Australia; 3 Priority Research Centre in Physical Activity and Nutrition, Faculty of Health, The University of Newcastle, Callaghan, 2308, NSW, Australia; 4 Environmental and Occupational Health and Safety, School of Health Sciences, Faculty of Health, The University of Newcastle, Callaghan, 2308, NSW, Australia; 5 School of Health Sciences, Faculty of Health and Priority Research Centre in Physical Activity and Nutrition, University of Newcastle, HA12 Hunter Building, University Drive, Callaghan, 2308, NSW, Australia

**Keywords:** Diet quality, Diet variety, Index, Score, Pediatrics, Child, Australia

## Abstract

**Background:**

Diet quality tools have been developed to assess the adequacy of dietary patterns for predicting future morbidity and mortality. This study describes the development and evaluation of a brief food-based diet quality index for use with children at the individual or population level. The Australian Child and Adolescent Recommended Food Score (ACARFS) was developed to reflect adherence to the Dietary Guidelines for Children and Adolescents in Australia and modelled on the approach of the US Recommended Food Score.

**Methods:**

The ACARFS has eight sub-scales and is scored from zero to 73. The diet quality score was evaluated by assessing correlation (Spearman’s correlations) and agreement (weighted κ statistics) between ACARFS scores and nutrient intakes, derived from a food frequency questionnaire in 691 children (mean age 11.0, SD 1.1) in New South Wales, Australia. Nutrient intakes for ACARFS quartiles were compared with the relevant Australian nutrient reference values.

**Results:**

ACARFS showed slight to substantial agreement (κ 0.13-0.64) with nutrient intakes, with statistically significant moderate to strong positive correlations with all vitamins, minerals and energy intake (r = 0.42-0.70). ACARFS was not related to BMI.Participants who scored less than the median ACARFS were more likely to have sub-optimal intakes of fibre, folic acid and calcium.

**Conclusion:**

ACARFS demonstrated sufficient accuracy for use in future studies evaluating diet quality. Future research on its utility in targeting improvements in the nutritional quality of usual eating habits of children and adolescents is warranted.

## Background

Diet quality is a recent dietary concept that refers to both the nutritional adequacy of individual’s dietary patterns, and how closely food patterns align with National Dietary Guidelines [1,2]. It also captures the complexity of food and nutrient combinations and interactions, as well as concepts of undernutrition and overnutrition [1,2]. The assessment of diet quality overcomes the limitations of linking intakes of single nutrients to health outcomes or disease risks [3]. Diet quality considers whole food and beverage intakes, which in turn are influenced by lifestyle behaviours, social determinants and the greater environment [1,4-9]. Chronic disease risk factors, including elevated systolic blood pressure, obesity and hyperglycaemia have been shown to be associated with poor diet quality in adults [1,10]. The validation of diet quality indices in relation to health outcomes in paediatric populations is difficult and controversial given the time lag in the development of chronic disease. However, paediatric diet quality indices have shown significant associations with, or have been validated using intermediate clinical markers for disease risk, including BMI [11,12], waist circumference [11,13], blood pressure [13,14], micronutrient intakes [12,15-18], and serum biomarkers such as iron, vitamin B_12_, and homocysteine [19,20].

Diet quality can be quantified as a single continuous variable through calculation of a diet quality index or score. The relationship between diet quality indices and nutritional adequacy and/or morbidity and mortality in adults has been reviewed [10] highlighting that across the 25 indices identified they are able to quantify risk for some health outcomes, including biomarkers of disease, incidence and risk of cardiovascular disease (CVD), some cancers and other chronic conditions and both cancer mortality and all-cause mortality [10].

However, these diet quality indices may be inappropriate for use in children due to age related differences in lifestyle, social influences, dietary intakes and dietary requirements [3]. To date no reviews of paediatric diet quality indices have been published, and only one diet quality index appropriate for the Australian context, the Dietary Guideline Index for Children and Adolescents (DGI-CA) [21] was found. The DGI-CA has 11 food based components and is derived from a 24 h recall implemented by trained interviewers [21]. The derivation of the final score requires nutrient analysis to calculate serves of food groups based on energy intake. The DGI-CA was found to be negatively associated with energy intake and although positive associations were found with BMI and waist-circumference z-scores in some age groups, they were considered weak with authors concluding that the DGI-CA was appropriate for use at the population level only [21].

Diet quality indices have been derived by applying a scoring system to dietary intakes assessed by food frequency questionnaires (FFQ), food records or 24 h recalls, with indices constructed by assigning higher scores within sub-scales based on higher intakes of foods, nutrients, or both [1]. Food based scores have some advantages over nutrient based scores as they rely on food and beverage consumption data only and thus can be scored quickly. For nutrient based scores, the dietary intakes must first be analysed to determine nutrient intakes before the overall score can be calculated. This suggests that food based scores may be more easily adapted for clinical and education purposes [1,22]. A food based diet diversity or variety score or diet quality index considers the number of foods or food groups consumed in a given period and reflects the relationship between dietary diversity and nutrient adequacy [1,2].

Therefore the aims of this study were to develop a paediatric diet quality index, the Australian Child and Adolescent Recommended Food Score (ACARFS) and to evaluate agreement with nutrient intakes from a previously validated FFQ as a measure of diet quality in children.

## Methods

### Study design and participants

The data in this study are from a cross-sectional survey of dietary intake, with anthropometric measurements collected from 720 primary school aged children (nine-12 yrs) from 29 randomly selected government schools in the Hunter Region of New South Wales (NSW), Australia in 2005 [23].

### Anthropometry

Body weight measurements were taken in light clothing without shoes using A&D Personal Precision Scale, UC 321, Accurate to 50 g (A&D Engineering, Inc. San Jose, California). Height was measured without shoes using a Harpenden Portable Stadiometer (98.603) classified as Class 1 under EC directive 93/42/EWG (Holtain Ltd. Crosswell, Crymych, UK-developed in collaboration with the Institute of Child Health at the University of London). Two measurements were taken for weight and height and the average calculated. A third measurement was taken for height and weight when the difference between the first two measurements was more than 0.5 centimetres or 0.5 kilograms, respectively, with the closest two measurements used to calculate the average. Average height and weight measures were used to calculate BMI using the formula: BMI = weight (kg)/[height (m)]^2^. Anthropometric data was transformed into BMI z-scores using the LMS method [24,25] based on reference data from the 1990 British Growth Reference [26].Weight status was classified as healthy weight, overweight or obese using the UK age and gender-specific BMI z-score cut-off points identified by Cole et al. [27] corresponding to a BMI of 25 for overweight and 30 for obese at 18 years of age.

### Assessment of dietary intake

Dietary intake was measured using the Australian Child and Adolescent Eating Survey (ACAES), a 135-item semi-quantitative FFQ with 120 food items and 15 supplementary questions addressing age, food behaviours and hours spent in sedentary behaviour. ACAES had been previously evaluated for reliability and relative validity and demonstrated acceptable accuracy for ranking nutrient intakes in Australian youth aged nine to 16 years [28-30]. Portion sizes for individual food items were accessed from the Australian Bureau of Statistics (ABS) [31] and unpublished data from the 1995 Australian National Nutrition Survey or the “natural” serving size for common items such as a slice of bread. Subjects were asked about frequency of their consumption over the previous 6 months. The frequency options ranged from ‘never’ to ‘4 or more times per day’ but up to ‘7 or more glasses per day’ for drinks. Of these frequency response questions, 24 related directly to intake of vegetables or legumes and 11 to fruit with seasonal availability of some fruits considered in the nutrient analysis, nine questions related to breads and cereals, nine to dairy foods, 32 to main meal or lunch items, nine to beverages, 20 to snack foods/dessert and six to sandwich spreads/dressing/ sauce. Nutrient intakes from the FFQ were computed from the most current food composition database of Australian foods available, the Australian AusNut 1999 database (All Foods) Revision 17 primarily and AusFoods (Brands) Revision five (Australian Government Publishing Service, Canberra) [32] to generate individual mean daily macro-and micronutrient intakes. The estimated daily intake for twenty macro- and micronutrients was calculated using FoodWorks (version 3.02.581 Xyris Software Australia, Highgate Hill, Queensland). In the current analysis FFQs that had greater than five unanswered items were excluded. For all FFQs included missing responses considered to be items that were never consumed.

This study was approved by the University of Newcastle Human Research Ethics Committee (approval number H-949-0205) and the New South Wales (NSW) Department of Education and Training. Written informed consent was obtained from all participants’ parents/guardians and participants gave informed assent prior to inclusion in the study.

### The Australian child and adolescent recommended food score

The ACARFS was designed as a brief, culture specific, food based diet quality tool for children and adolescents. It focused on dietary variety within recommended food groups for use in populations and individuals and took 10 to 15 minutes to complete. It was modelled on the Recommended Food Score [33] and the Australian Recommended Food Score (ARFS) [34] which evaluated adherence to National Dietary Guidelines for Adults. The ACARFS utilises a subsample of questions in the ACAES FFQ [28] which are consistent with the eating patterns recommended in the Australian Dietary Guidelines for Children and Adolescents [35]. The ACARFS has eight food group components, seventy questions and a score ranging from zero to 73 (Table 1) with 20 questions related directly to vegetable intake, 12 to fruit, 13 to protein foods (seven to meat and six to non-meat protein foods), 12 to breads/cereals, 10 to dairy foods, one to water and two to spreads/sauce. The scoring for each food group component is detailed in Table 1.The maximum possible score for each component was determined by the number of suitable ACAES FFQ items in each food group with scoring based on how frequently the included items were consumed. Most foods were awarded one point for a reported consumption of ≥ once per week, but differed for some items depending on national dietary guideline recommendations [35] with consideration of the Australian Guide to Healthy Eating [36]. Some food items had a limit placed on their score for higher intakes due to higher intakes being associated with potentially higher saturated fat or disease risk and these capped items received only one point for the following; minced meat consumed > never to < once per month; beef and/or lamb, chicken without crumbing or batter or pork consumed one to four times per week; flavoured milk, ice-cream or frozen yoghurt consumed ≥ once per week to ≤ once per day; cheese, cheese spread or cream cheese consumed ≥ once per week but < four per day; and water if consumed ≥ four glasses per day. A scoring cap was applied to meats as only 0.5 to 1 serve of meat (including fish and poultry) is recommended per day in national guidelines for children and adolescents [35,36]. Some dairy foods including flavoured milk, ice-cream, frozen yoghurt and cheese were capped due to their high saturated fat and/or sugar content [35,37]. Additional points were awarded (Table 1) for consuming evening meals with vegetables ≥ three times per week, ≥ one pieces of fruit per day, ≥ two serves of milk, yoghurt or cheese per day, and for using reduced fat milk and wholegrain or wholemeal bread. The type of fat spreads was not asked as children are unlikely to know this. Yeast extract spread and tomato ketchup were included in the score as they contain a significant amount of B-group vitamins or β-carotene respectively [37].

**Table 1:** Scoring method for items in the Australian Child and Adolescent Recommended Food Score

**Food Group**	**Items giving 1 point**	**Items giving more than 1 point**	**ACARFS**
Vegetables	3-4 nightly meals with vegetables‡; ≥1per week of each of the following vegetables: potato, pumpkin, sweet potato, cauliflower, green beans, spinach, cabbage or Brussels sprouts, peas, broccoli, carrots, zucchini or eggplant or squash, capsicum, corn, mushrooms, tomatoes, lettuce, celery or cucumber, avocado, onion or leek or shallots/spring onion.	2 points for ≥5 nightly meals with vegetables^‡^	21
Fruit	≥1 piece of fruit per day, ≥1 per week of each of the following fruit: canned fruit, fruit salad, dried fruit, apple or pear, orange or mandarin or grapefruit, banana, peach or nectarine or plum or apricot, mango or paw-paw, pineapple, grapes or strawberries or blueberries, melon (any variety).		12
Protein Foods – Meat/flesh	≤1 serve of mince meat per month but greater than never; 1 – 4 serve per week of: beef or lamb with or without sauce and/or vegetables per week chicken without batter or crumbing but with or without sauce and/or vegetables, pork with or without sauce and/or vegetables; ≥1 per week of fresh fish, canned tuna or salmon or sardines, other seafood (e.g. prawns, lobster).		7
Protein Foods – Meat/flesh alternatives	≥1 per week of the following: nuts (e.g. peanuts, almonds), nut butters, eggs, soybeans or tofu, baked beans, other beans or lentils (e.g. chickpeas, split peas).		6
Grains	Usual bread choice is ‘other’ (e.g. rye, high-fiber white); ≥1 per week of the following: muesli, cooked porridge, breakfast cereal (e.g. Weet-bix, Nutri-grain, Cornflakes), bread or pita bread or toast, English muffin or bagel or crumpet, rice, other grains (e.g. couscous, burghul), noodles (e.g. egg noodles, rice noodles), pasta, tacos or burritos or enchiladas, clear soup with rice or noodles.	2 points if usual bread choice is ‘brown’ (multigrain or wholemeal).	13
Dairy	≥2 serves of: milk, yoghurt or cheese per day; ≥1 serve per week but ≤1 serves per day of flavoured milk, ice cream, frozen yoghurt; ≥1 serve per week but ≤4 serves per day of cheese, cheese spread or cream cheese; ≥1 serve per week of plain milk, yoghurt (not frozen), cottage cheese or ricotta.	2 points if usual type of milk is reduced fat milk or skim milk, or soy milk	11
Water	≥4 glasses of water (including tap, unflavoured bottled water, unflavored mineral water).		1
Extras	≥1 serve per week of: yeast extract spread; tomato or barbecue sauce		2
**Total**			**73**

‡Although the serves of vegetables consumed each day is of interest, the question was not available on the ACAES FFQ.

### Confounding factors

Health-related variables potentially associated with food intake and adjusted for in the multiple regression were gender, hours spent in sedentary behaviour, BMI z-score [38] and age. Meeting sedentary behaviour recommendations (<0–1 h spent watching television and <0–1 h spent at the computer or playing video games), height, weight, BMI category, gender, school year, and Socio-economic status (SES) were also described. SES was assessed using the Socio-Economic Index for Areas (SEIFA) [39] at the level of the school, based on school postcode, but was not included in the regression as it was not available at the individual level.

### Statistical analysis

Health-related variables were described for the whole group and by school year. Correlation and multiple regression analysis were used to test for significant associations with the potential confounders: age, BMI z-scores and hours spent in sedentary activity. Descriptive statistics were used to summarize the ACARFS for each school year and by gender and also to describe the contribution of each of the core food component scores to the total ACARFS. Normality of data was tested using the Shapiro-Wilk test, the χ^2^ test was used for testing association between categorical variables, the Two Sample t-tests (two-tailed) for parametric continuous variables, and the Wilcoxon test (two-tailed) for non-parametric continuous variables.

The weighted kappa (κ) statistic, a quantitative measure of agreement where chance is accounted for, was used to assess agreement between ACARFS quartiles with quartile distributions of the average daily intake of 13 nutrients: percent energy from saturated fatty acids (SFA), fibre, vitamin C, vitamin A, β-carotene, thiamin, riboflavin, niacin, folate, calcium, magnesium, iron and zinc. Quartile four represented the highest ACARFS score or highest intake of each nutrient except for percent energy from (saturated fatty acid) SFA in which quartile four represented the lowest intake. The macronutrients of total fat, mono-unsaturated fatty acids (MUFA), poly-unsaturated fatty acids (PUFA), protein and carbohydrate were not assessed using the weighted κ statistic as there is no definite recommendation for increasing or decreasing their intake, for example the recommended fat intake is 30% whereas SFA intake is less than 10% [40]. The weighted κ statistic was chosen to compare ACARFS scores with nutrient intakes as it summarizes the agreement in categories of ranked ACARFS scores in reference to each nutrient assessed from the FFQ. This provided information to assess the usefulness of the ACARFS in individuals as well as populations. In addition, the non-weighted κ statistic was calculated for each ACARFS quartile and its corresponding quartile of nutrient intake to further investigate their agreement. Kappa values of 1.0 represent perfect agreement, 0.0 represents no more agreement than would occur due to chance and negative values suggest there is less agreement than would occur due to chance alone [41]. Arbitrary benchmarks for κ statistics have been set as 0.01 – 0.20 ‘slight’ agreement, 0.21 – 0.40 ‘fair’, 0.41–0.60 ‘moderate’, 0.61–0.80 ‘substantial’ and 0.81–0.99 ‘almost perfect’ agreement [42].

However, the κ statistic may be an overly conservative assessment of agreement as the degree of chance is thought to be overestimated [43]. To further explore the relationship between the ACARFS and nutrient intakes determined from the ACAES FFQ, Spearman’s correlation coefficients (as the nutrient intakes were skewed to the left) were calculated for the 13 nutrients listed previously as well as energy and percentage of energy from protein, carbohydrate, fat, SFA, MUFAs, PUFAs and sugar. Descriptive statistics were used to evaluate nutrient intakes by ACARFS quartile and in comparison to the Nutrient Reference Values (NRVs) of Recommended Dietary Intakes (RDIs) or Adequate Intakes (AI, used when the RDI cannot be determined), where appropriate, for children aged nine to 13 years [40]. The RDI was chosen as the benchmark for comparison as it is the average daily intake level sufficient to meet the needs of 97 to 98% of the population, a more ambitious target than the Estimated Average Requirement (EAR) which is the level required to meet the needs of only 50% [40]. The RDI was used to indicate the ACARFSs association with nutrient intakes.

For the analysis, a p-value of < 0.05 was considered statistically significant. Statistical analysis was undertaken using STATA version 8, StataCorp LP, College Station, Texas (2003) except for the unweighted kappa statistics which were evaluated using JMP v 8, SAS Institute Inc, Cary, North Carolina (2009) as this function was unavailable in STATA version 8.

## Results

### Study participants

The demographic characteristics of participants (*n* 720) are reported in Table 2. Boys had lower mean BMI z-score compared to girls (0.57 ± 1.16 versus 0.75 ± 1.18, *P =* 0.040). Twenty-nine (four percent) of participants were found to have five or more unanswered ACAES FFQ items and were therefore excluded. These excluded participants (*n* 29) were not significantly different (*P* > 0.05) from the remaining participants (*n* 691) in any of the variables considered in Table 2.

**Table 2:** Anthropometric and socio-demographic data of the excluded and remaining participants in the ACARFS study

	**Excluded Participants (*n*29)**	**Remaining participants**		
		**Total (*n* 691)**	**Year 4 (*n* 354)**	**Year 6 (*n* 337)**
School Year 4 (%)	58.6*	51.2*		
Female (%)	65.5*	56.2*	56.2	56.1
Age (years) -mean ± SD	10.8 (±1.1)^†^	11.0 (±1.1)^†^	10.0 (±0.4)	12.1 (±0.4)
Height (cm) -mean ± SD	146.8 (±7.7)^†^	145.7 (±9.4)^†^	139.4 (±6.7)	152.4 (±6.9)
Weight (kg) -mean ± SD	40.9 (±7.4)^†^	41.0 (±10.8)^†^	35.9 (±8.3)	46.3 (±10.6)
BMI (kg/m^2^) -mean ± SD	18.9 (±2.6)^†^	19.1 (±3.5)^†^	18.4 (±3.2)	19.8 (±3.5)
BMI z-score -mean	0.73 (±1.1)^†^	0.67 (±1.2)^†^	0.63 (±1.2)	0.72 (±1.1)
Overweight (%)^a^	20.7^†^	22.3†	21.2	23.4
Obese (%)^a^	6.9^†^	5.8^†^	6.5	5.1

cm, centimetres; m, meters; SD, standard deviation. ^a^ Healthy weight, overweight or obese classified using the UK age and gender-specific BMI z-score cut-off points that correspond to a BMI of 25 for overweight and 30 for obese at 18 years of age. * *P*-values for the comparison of excluded participants and remaining participants using the students t-test (two-tailed) were not significant (*P* > 0.05). †*P*-values for the comparison of excluded participants and remaining participants using the Wilcoxon test (two-tailed) were not significant (*P* > 0.05).

### Australian child and adolescent recommended food score

The ACARFS was calculated for 691 children and was slightly skewed to the left. Table 3 reports the ACARFS descriptive statistics overall, by gender and school year. From a possible maximum score of 73, the median ACARFS score was 25 with a maximum of 58 and a minimum of three. Females and older primary school students (mean age ≈ 12 years) had a significantly higher mean ACARFS than males and younger children (mean age ≈ 10 years) respectively (*P <* 0.001, *P =* 0.005). Table 4 reports the contribution of each ACARFS component to the overall score.

**Table 3:** The Australian Child and Adolescent Recommended Food Score (ACARFS) overall and by gender and school year

			**Gender**		**School Year**	
	**Total (*n* 691)**	**Female (*n* 388)**	**Male (*n* 303)**	**Year 4 (*n* 354)**	**Year 6 (*n* 337)**	
Median	25	26	24	24	26
25^th^ percentile	19	20	17	17	20
75^th^ percentile	32	33	30	31	32
IQR	13	13	13	14	12
Min score	3	5	3	3	3
Max score	58	55	58	58	55
Range	55	50	55	55	52

IQR, inter-quartile range; Min, minimum; Max, maximum.

**Table 4:** **Australian Child and Adolescent Recommended Food Score (ACARFS) and component scores as calculated for children (*n *691) from New South Wales**

**Component score (max number possible)**	**Median**	**IQR**	**Min**	**Max**
Vegetables (21)	7	6	0	20
Fruit (12)	5	5	0	12
Protein Foods – Meat/Flesh (7)	2	2	0	6
Protein Foods – Meat/Flesh Alternatives (6)	1	1	0	6
Grains (13)	4	3	0	11
Dairy (11)	4	3	0	9
Water (1)	1	1	0	1
Extras (2)	1	1	0	2
Total ACARFS (73)	25	13	0	58

ACARFS, Australian Child and Adolescent Recommended Food Score; Max, maximum; Min, minimum.

### Confounding factors

The correlation between BMI z-score and the ACARFS was not significant (r = 0.02, *P =* 0.610). Multiple linear regression showed that together gender, BMI z-score, age and hours/day spent in sedentary pursuits explained a small amount of the variation in the ACARFS (R^2^ = 0.04, *P* < 0.001). Individual regression coefficients were gender = 0.1, BMI z-score = 0.03, age = 0.10 and sedentary behaviour = 0.12. Twenty-six percent of the participants met the recommendations for minimizing sedentary behaviour with girls more likely than boys (*P <*0.001) but the difference by school year was not significant (*P >* 0.05). Thirty-four percent of children reported that they spent ≥ 4 h/d in sedentary activity, and five percent reported ≥ 8 h/d. The SEIFA codes for all schools were below the NSW average for the Socio-Economic Index for Areas of disadvantage [28,39].

### Assessment of agreement

The ACARFS quartiles were assessed for agreement with quartiles of nutrient intakes (Table 5). The percentage of ACARFS scores and nutrient intakes classified into the same quartile, the percentage classified in the same or adjacent quartile, and the percentage grossly misclassified (i.e. those classified as quartile four for ACARFS but classified as quartile one for nutrients and vice-versa) were calculated. The κ statistic for each quartile was calculated as was the overall weighted κ statistic, standard error and p-value. The percent energy intake from SFA gave the least overall agreement of all the nutrients (κ = 0.13) and demonstrated ‘slight’ agreement, followed by riboflavin (κ = 0.36) which showed ‘fair’ agreement. Vitamin C (κ = 0.64), fibre (κ = 0.62) and β-carotene (κ = 0.62) had the strongest ‘substantial’ agreement. All other nutrients showed ‘moderate’ agreement (κ = 0.42 – 0.56). Within quartiles, fibre, vitamin C and β-carotene had the lowest percentages grossly misclassified. With the exception of SFA, all other nutrients had less than five percent grossly misclassified. The strongest agreement amongst the quartiles was quartile one, where nine of the nutrients showed ‘moderate’ agreement. Quartile four showed the next strongest agreement with agreement in quartiles two and three rated as very slight.

**Table 5:** Comparison of nutrient intakes as assessed by the Australian Child and Adolescent Eating

**Nutrients**	**ACARFS**									
	**Percent classified in the same quartile**	**Percent classified in the same or adjacent quartile**	**Percent grossly misclassified**	**Quartile 4 κ**^**‡**^	**Quartile 3 κ**^**‡**^	**Quartile 2 κ**^**‡**^	**Quartile 1 κ**^**‡**^	**Overall weighted κ**	**Overall strength of agreement**^**§**^	**Spearman’s correlation coefficient**
% energy from SFA	28.4	66.2	9.4	0.06	−0.01	−0.01	0.13	0.13^∥^*	Slight	−0.15***
Fiber	47.5	87.2	1.2	0.36	0.12	0.16	0.56	0.62^∥^**	Substantial	0.67***
Thiamin	41.4	79.0	4.3	0.24	0.15	0.09	0.39	0.43^∥^**	Moderate	0.47***
Riboflavin	36.9	76.6	5.6	0.14	0.10	0.05	0.35	0.36^∥^**	Fair	0.42***
Niacin	41.3	82.3	3.3	0.27	0.09	0.09	0.41	0.49^∥^**	Moderate	0.56***
Folate	43.1	83.5	3.3	0.28	0.16	0.09	0.44	0.51^∥^**	Moderate	0.56***
Vitamin C	47.0	89.0	0.9	0.39	0.16	0.13	0.50	0.64^∥^**	Substantial	0.70***
Vitamin A	36.3	78.3	3.2	0.15	0.03	0.02	0.40	0.43^∥^**	Moderate	0.49***
Β-Carotene	46.6	87.1	0.6	0.35	0.11	0.13	0.56	0.62^∥^**	Substantial	0.67***
Iron	44.0	82.2	3.2	0.28	0.15	0.10	0.48	0.50^∥^**	Moderate	0.54***
Magnesium	45.7	84.6	2.2	0.33	0.12	0.17	0.49	0.56^∥^**	Moderate	0.62***
Calcium	39.4	78.6	4.9	0.22	0.11	0.08	0.36	0.42^∥^**	Moderate	0.46***
Zinc	40.1	82.1	2.3	0.28	0.02	0.08	0.42	0.50^∥^**	Moderate	0.56***

Survey (ACAES) versus Australian Child and Adolescent Recommended Food Score (ACARFS). ACAES, Australian Child and Adolescent Eating Survey; ACARFS, Australian Child and Adolescent Recommended Food Score; κ, kappa statistic; SFA, saturated fatty acids. ‡Quartile 4 indicates the highest ACARFS (32–58) and nutrient intakes, quartile 3 indicates the second highest ACARFS (26–31) and nutrient intakes, quartile 2 indicates the second lowest ACARFS (19–25) and nutrient intakes, quartile 1 indicates the lowest ACARFS (3–18) and nutrient intakes. § Landis and Koch Classification [42]. ∥Standard Error: 0.04 *P < 0.0005 **P < 0.001 *** Significantly different from 0, *P* < 0.001.

### Correlation

ACARFS demonstrated statistically significant positive correlations with all vitamins and minerals tested (Table 5). The strongest correlations were with vitamin C (r = 0.70, *P* < 0.001), β-carotene (r = 0.67, *P* < 0.001) and fibre (r = 0.67, *P <* 0.001). ACARFS also had a moderately strong positive correlation with total energy (r = 0.51, *P <* 0.001). When the ACARFS was correlated with macronutrients adjusted for energy intake there was a weak positive correlation with protein (r = 0.18, *P <* 0.001) and weak negative correlation with total fat (r = −0.12, *P =* 0.003) and SFA (r = −0.15, *P <* 0.001). Associations between the ACARFS and percent energy intake from MUFA, PUFA, carbohydrate and sugar intake were not significant.

### Nutrient reference values

Table 6 describes the NRVs appropriate for individual children aged nine to 12 for the nutrients considered in the analysis, and the median and IQR of nutrient intakes as calculated by the ACAES FFQ for each of the ACARFS quartiles. In both quartile three and quartile four (highest ACARFS score) all of the median nutrient intakes met the corresponding NRV. In both quartile one (lowest ACARFS quartile) and quartile two the median nutrient intakes of the population sample for fibre, folate and calcium did not meet the corresponding NRV. Table 6 also shows the proportion of the sample population not meeting the corresponding NRV. This was the greatest for fibre (45%), folate (45%) and calcium (40% <1000 mg, 62% <1300 mg) and the lowest for niacin equivalents (1%) and riboflavin (0.3%).

**Table 6:** **Comparison of nutrient intakes of the study population (*n*691) with nutrient reference values**

**Nutrients**	**NRV (RDI/AI)**^**(31)**^	**Quartile 4**		**Quartile 3**		**Quartile 2**		**Quartile 1**		**% participants not meeting the NRV**
		**Median**	**IQR**	**Median**	**IQR**	**Median**	**IQR**	**Median**	**IQR**	
Fibre	24 g (AI)	35 g	16	29 g	12	23 g	13	16 g	10	45
Thiamin	0.9 mg (RDI)	2.3 mg	1.2	2.1 mg	0.9	1.7 mg	1.1	1.3 mg	0.9	8
Riboflavin	0.9 mg (RDI)	3.4 mg	1.8	3.1 mg	1.6	2.5 mg	1.7	1.9 mg	1.6	0.3
Niacin	12 mg (RDI)	53 mg^‡^	24	47 mg^‡^	20	38 mg^‡^	19	28 mg^‡^	18	1
Folate	300 μg (RDI)	412 μg	176	353 μg	147	296 μg	152	215 μg	120	45
Vitamin C	40 mg (RDI)	190 mg	98	144 mg	74	112 mg	70	71 mg	55	6
Vitamin A	600 μg (RDI)	1710 μg^§^	1066	1652 μg	1128	1273 μg	997	733 μg	828	12
Iron	8 mg (RDI)	17 mg	8	15 mg	7	13 mg	6	10 mg	6	12
Magnesium	240 mg (RDI)	471 mg^∥^	150	396 mg^∥^	144	340 mg	125	259 mg	130	12
Calcium	1000-1300 mg (RDI) ^¶^	1436 mg	720	1270 mg^¶^	535	998 mg	581	803	522	40-62
Zinc	6 mg (RDI)	17 mg	7	14 mg	7	12 mg	6	9 mg	6	7

NRV, Nutrient Reference Value; ACAES, Australian Child and Adolescent Eating Survey; RDI, Recommended Dietary Intake; AI, Adequate Intake; IQR, inter-quartile range; eq, equivalent. **‡** The median intake of niacin is above the upper limit of 20 mg/day [40]. § The median intake of vitamin A is above the upper limit of 1700 μg/day if the total vitamin A source was from retinol, however the vitamin A (retinol equivalents) estimated intake in the sample population includes β-carotene which is not known to result toxicity [40]. ∥ The median intake of magnesium is above the upper limit of 350 mg/day [40]. A median calcium intake of 1270 mg does not meet the RDI for children aged 12 to 13 years and children aged nine to 11 years who are growing at a greater rate than average. The RDI for these groups is 1300 mg [40].

## Discussion

National Dietary Guidelines for children and adolescents recommend that children enjoy a wide variety of nutritious foods [35]. The ACARFS was designed to capture eating habits and food behaviours recommended within these guidelines. Therefore, like the adult Recommended Food Score [33,34] it only considers intake of foods that align with dietary guidelines. Although the median score was not high at 25, the ACARFS correlated with nutrient intakes in the direction expected and applying Kappa statistics to quartiles of score, generally agreed with estimated nutrient intakes assessed from the ACAES FFQ. Importantly, children with higher ACARFS scores were more likely to meet the NRVs. The ACARFS therefore implies one or both of the following; firstly that a child who scores well on the ACARFS consumes a wide variety of *healthy* foods and has an adequate nutrient intake, or secondly that a child who scores well on the ACARFS consumes a wide variety of *healthy* and *unhealthy* foods, but still has an adequate nutrient intake. It is important to note that the dietary guidelines are not disease specific. Therefore adherence to the guidelines may have a varied effect on chronic disease risk and no assumptions can be made about higher ACARFS scores and decreased risk of chronic diseases without research to specifically evaluate this.

Strategies to enhance diet quality should potentially focus on promotion of a greater variety of sources of lean protein food, high fibre and wholegrains, vegetables and reduced fat dairy foods. Our results indicate that the majority of individuals were categorised within one quartile for both the ACARFS and the majority of nutrients evaluated, and rated as having moderate to substantial agreement. This is encouraging in terms of using a diet quality tool to potentially evaluate the impact of a broad public health campaign aimed at promoting adherence to national dietary guidelines or improving overall diet quality.

When applied to the quartiles of ACARFS and nutrient intakes, the weighted κ statistics showed slight to substantial agreement overall, though most nutrients showed moderate agreement [42]. This indicates that the ACARFS is moderately strong in correctly classifying an individual or populations as having either good diet quality or poor diet quality. However the ability to correctly classify those in middle quartiles is slight. This could be partly explained by the non-normal data with skews to lower values for the ACARFS and nutrient intakes. Furthermore, the ACARFS correctly classified a third to almost half of individuals into the same quartile for nutrient intakes, with the exception of percent energy from SFA. The strongest agreement between ACARFS and nutrient intake was for vitamin C, fibre, β-carotene and magnesium, where the majority of individuals were classified into the same or adjacent quartile. The poorest agreement was for percent energy from SFA where the weighted κ statistic was slight but two thirds of individuals were classified into the same or adjacent quartile. For SFA the correlation with ACARFS produced a similar result with a slightly negative and statistically significant correlation with percent energy from SFA. The modest results regarding SFA may be due to the dairy and/or meat/flesh components of the ACARFS as many of these foods may contain large amounts of SFA, such as cheese or red meats [37]. While correlation with all of the vitamins and minerals was moderately strong and statistically significant, correlation with fibre, vitamin C, β-carotene and magnesium were strongest. The ACARFS was also positively correlated with energy intake, a common finding in variety indices such as the ACARFS or dietary diversity scores, as the more food consumed the more variety in the diet and the higher the nutrient intakes [44-46]. Although this relationship with energy exists and diet quality and variety scores have been known to be positively associated with BMI [21,47], the ACARFS was not correlated with BMI, and BMI z-scores had minimal influence on variation in the ACARFS.

Although participants, with the lowest ACARFS scores, indicating the poorest diet quality had the lowest nutrient intakes of the sample population, they still met most of the RDIs and AIs. However, the NRVs for fibre, folate and calcium were not met by about half of the participants in quartile one or two. This indicates that the ACARFS is sensitive enough to identify participants not eating a sufficient variety of nutrient rich foods. Even those participants not eating a wide variety of nutritious foods are unlikely to be deficient in the other vitamins and minerals considered as these nutrients are plentiful in the Australian food supply [37,40]. It is important to note that the ACARFS is determined by the number of foods from each food group usually consumed at least weekly. This means that although an individual may consume the recommended servings of each food group, such as one fruit and three vegetables each day [36], which would provide a sufficient intake of most nutrients, consumption of a wide variety from each food group every week is required to gain a high score.

### Limitations

The sample population were aged nine to 12 years only and had a lower SES than the NSW average which may reduce how generalisable it is to other populations. While parents can fill in the ACARFS on behalf of their child, this may introduce bias as parents have been reported to overestimate child diet quality [48]. The relative contribution of each component to the final score was dependent on the questions in the ACAES FFQ and is not necessarily representative of the Australian Guide to Healthy Eating [36]. However, this may be viewed as a strength and a more realistic representation of the food group proportions available in the food supply. As the dietary guidelines for children in Australia do not provide specific recommendations for amounts to be consumed within food groups, the scoring contains an additional degree of subjectivity. This potentially means the ACARFS could overestimate usual diet quality. Further, given that biomarkers to objectively verify components of dietary intake were not measured, the results should be interpreted with caution.

In the ACAES validation study the FFQ demonstrated higher nutrient intakes compared to food records [28]which may explain why the median intakes of niacin, vitamin A and magnesium were above the corresponding upper limit. However, the ACAES FFQ validation study demonstrated the ability of the ACAES FFQ to correctly classify participants into quintiles of nutrient intake and therefore not affect the assessment of agreement and correlations [28]. This also suggests that participants in the first and second ACARFS quartiles may be at risk of inadequate intakes of nutrients other than fibre, folate and calcium.

### Implications for research and practice

As the ACARFS is derived from a validated FFQ for children it offers the opportunity for researchers to use it independently or to derive it secondarily from the FFQ as a measure of overall dietary quality as a single continuous variable. The calculation of the ACARFS from the full ACAES FFQ is less onerous than indices that include nutrient based sub-scales. Its use as a brief tool to assess diet quality using only the FFQ questions and relevant responses could extend its use by allowing the ACARFS to be used along with the provision of timely feedback.

To extend its usability further research should examine use of the ACARFS method applied to other FFQs, in other populations, age groups, as well as other settings such as a self-monitoring tool or within clinical practice. In order for the ACARFS to be of use clinically or for self-assessment, then cut points may need to be derived. However, the agreement between quartiles suggests that those with an ACARFS score of 32 and above have a good diet quality and consume a reasonably wide variety of nutritious foods and that they have the highest nutrient intakes. Those with an ACARFS score of 19 to 31 (quartiles two and three) have a moderate diet quality, and consume a moderate variety of nutritious foods, but are at risk of sub-optimal intakes of fibre, folate and calcium. Finally, those with an ACARFS score of 18 or less have a poorer diet quality, do not consume a wide variety of nutritious foods and have the lowest intakes of a range of nutrients.

## Conclusion

The ACARFS is a brief assessment tool to measure the diet quality, food variety and nutritional adequacy of dietary intakes of Australian youth. Based on the correlation analyses and weighted κ statistics to assess agreement with ranked nutrient intakes presented for this sample of children the ACARFS may be a useful tool in evaluating the diet quality of individuals and populations. Future research is needed to identify whether the ACARFS can be used effectively to target improvements in diet quality within community and within clinical interventions aimed at optimising the dietary intakes of children and adolescents.

## Abbreviations

ABS: Australian bureau of statistics; ACAES: Australian child and adolescent eating survey; ACARFS: Australian child and adolescent recommended food score; AI: Adequate intakes; ARFS: Australian recommended food score; BMI: Body mass index; CVD: Cardiovascular disease; DGI-CA: Dietary guideline index for children and adolescents; EAR: Estimated average requirement; FFQ: Food frequency questionnaire; MUFA: Mono-unsaturated fatty acids; NRV: Nutrient reference values; NSW: New South Wales; PUFA: Poly-unsaturated fatty acids; RDI: Recommended dietary intakes; SEIFA: Socio-economic index for areas; SES: Socio-economic status; SFA: Saturated fatty acids.

## Competing interests

The authors declare that there are no competing interests. This study was conducted at the University of Newcastle, Callaghan, NSW, AUSTRALIA and received no specific grant from any funding agency in the public, commercial or not-for-profit sectors. CECis supported by an Australian National Health and Medical Research Council Career Development research fellowship.

## Authors’ contributions

All authors contributed to the research design and interpretation of results, SM conducted the primary analysis with assistance from JW. SM created the first manuscript draft and all authors revised the manuscript and approved the final version. The contribution of SM was conducted as part requirement for the degree of Bachelor of Nutrition and Dietetics (Honours).
